# Nasopharyngeal carriage of *Streptococcus pneumoniae* among children and their household members in southern Mozambique five years after PCV10 introduction

**DOI:** 10.1016/j.vaccine.2024.126691

**Published:** 2025-02-15

**Authors:** Rebecca Kahn, Benild Moiane, Fernanda C. Lessa, Sergio Massora, Viviana Mabombo, Alberto Chauque, Nelson Tembe, Helio Mucavele, Cynthia G. Whitney, Charfudin Sacoor, Graca Matsinhe, Fabiana C. Pimenta, Maria da Gloria Carvalho, Betuel Sigauque, Jennifer Verani

**Affiliations:** a Respiratory Diseases Branch, Division of Bacterial Diseases, Centers for Disease Control and Prevention, Atlanta, United States; b Centro de Investigação em Saúde da Manhiça (CISM), Maputo, Mozambique; c International Infection Control Branch, Division of Healthcare Quality Promotion, Centers for Disease Control and Prevention, Atlanta, United States; d Instituto Nacional de Saúde, Ministério de Saúde, Maputo, Mozambique; e Emory University, Atlanta, United States; f National Expanded Program on Immunization - Ministry of Health, Mozambique; g John Snow Inc (JSI) on the MOMENTUM Routine Immunization Transformation and Equity (M-RITE), Maputo, Mozambique

**Keywords:** Pneumococcal conjugate vaccines, Mozambique, Carriage, Antimicrobial resistance, Vaccine impact

## Abstract

**Background:**

*Streptococcus pneumoniae* is an important cause of pneumonia, sepsis, and meningitis, which are leading causes of child mortality. Pneumococcal conjugate vaccines (PCVs) protect against disease and nasopharyngeal colonization with vaccine serotypes, reducing transmission to and among unvaccinated individuals. Mozambique introduced 10-valent PCV (PCV10) in 2013. In 2017–2019, 13-valent PCV (PCV13) replaced PCV10, and in September 2019 the schedule changed from three primary doses to two primary doses and a booster; the booster-containing schedule may increase indirect effects. We examined pneumococcal carriage in Mozambique to establish a baseline for estimating the impact of policy changes and to estimate the long-term impact of PCV10 in children aged <5 years.

**Methods:**

We calculated prevalence of carriage of PCV10 serotypes and the 3 additional PCV13 serotypes (‘PCV13-unique’) among children aged <5 years and their household members in southern Mozambique, between October 2018 and July 2019. Nasopharyngeal swabs were cultured, and isolates underwent Quellung serotyping. For children, we compared these “long-term post-PCV10” data with prior surveys (“pre-PCV” (2012−2013) and “post-PCV10” (2015–2016)) that used the same methods.

**Results:**

In 2018–2019, among 1319 children aged under five years, 1064 (80.7 %) were colonized with pneumococcus, among 614 children aged 5- < 18 years, 355 (57.8 %) were colonized, and among 804 adults (aged ≥18 years), 285 (35.4 %) were colonized. The most frequently observed serotypes were 19 A (*n* = 154, 8.5 % of isolates) and 6 A (*n* = 107, 5.9 %), both PCV13-unique serotypes. Overall carriage prevalence among children under five years remained stable at approximately 80 % across the carriage studies conducted between 2012 and 2019; between 2015 and 2016 and 2018–2019, the prevalence of PCV10-type carriage declined from 17.7 % to 10.1 %.

**Conclusions:**

Despite substantial declines in PCV10-type carriage initially following vaccine introduction, the continued circulation of PCV10 serotypes and relative high prevalence of PCV13-unique serotypes underscore the need to understand the impact of policy changes on pneumococcus transmission.

## 1 Introduction

*Streptococcus pneumoniae* (pneumococcus) is a major cause of disease worldwide [1]. In recent years, an estimated 300,000 children <5 years of age die of pneumococcal diseases annually, with the majority of deaths occurring in sub-Saharan Africa and Asia [2,3]. Pneumococcal colonization is a precursor to disease [4,5], which occurs when pneumococcus spreads from the nasopharynx to other organs such as the lungs, bloodstream, and meninges, causing pneumonia, sepsis, and meningitis, respectively [4]. Healthy children under five years of age are the main reservoirs of pneumococcus and therefore play an important role in transmission [4]. An estimated 27–85 % of children are pneumococcal carriers, with higher rates in lower income countries [6].

Pneumococcal conjugate vaccines (PCVs) provide protection against several of the over 100 serotypes of *S. pneumoniae* bacteria. Serotypes included in the vaccines are among those that cause the most morbidity and mortality. Currently the two most widely used formulations provide protection against 10 (Synflorix, GlaxoSmithKline product [PCV10]) and 13 (Prevnar13, Pfizer [PCV13]) serotypes, while recently licensed formulations for use in children contain up to 15 or 20 serotypes [7]. A relatively less expensive 10-valent PCV (Pneumosil, Serum Institute of India [PCV10-SII]) that protects against two of the serotypes covered by PCV13 has been available since 2020 [8]. In addition to protecting individuals who receive the vaccines (i.e., direct effects), PCVs prevent carriage and spread of vaccine serotypes, protecting the broader community (i.e., indirect effects) [6,9,10]. Carriage studies, which assess prevalence of colonization, can provide important information around circulating serotypes, antimicrobial susceptibility, and community-wide impact of PCVs [11].

Mozambique introduced PCV10 on April 10, 2013, with support from 10.13039/100001125Gavi, the Vaccine Alliance, using a 3-primary dose schedule (at 2, 3, and 4 months of age; “3 + 0”). Analysis of repeated cross-sectional carriage studies conducted in Mozambique found that within 2–3 years after introduction, PCV10-type carriage had declined by approximately 50 % in children under five years of age [12]. However, PCV10-type serotypes continued to circulate, and serotype 19 A (included in PCV13 but not in PCV10) was increasingly common [12]. Beginning in 2017, Mozambique switched from PCV10 to PCV13 formulation for new birth cohorts, using a phased approach starting in the northern part of the country, and reaching the southern region on May 5, 2019. Additionally, on September 23, 2019, Mozambique changed the PCV dosing schedule nationwide from the original “3 + 0” schedule (three primary doses without a booster) to a “2 + 1” schedule (two primary doses at two and four months followed by a booster at nine months of age), which provides vaccinated children higher levels of antibodies in the second year of life, potentially contributing to more robust direct and indirect effects [13].

We conducted a cross-sectional carriage survey among children aged <5 years and their household contacts in southern Mozambique from October 16, 2018 to July 4, 2019, during which time PCV coverage in Mozambique was estimated to be 80–95 % [14], to provide a baseline for evaluating the impact of the PCV schedule and formulation changes and to assess the longer-term impact of PCV10 introduction.

## 2 Methods

### 2.1 Study site and population

This study was carried out from October 16, 2018 to July 4, 2019, using the same methods as three prior cross-sectional surveys conducted before (October 29, 2012 to April 16, 2013) [15] and after (October 6, 2014 to April 10, 2015; October 6, 2015 to May 24, 2016) PCV10 introduction [12,16] in Mozambique. The survey took place in southern Mozambique in one urban (Mavalane) and one rural (Manhiça) area, which were both included in the previous surveys; Nampula, an urban area in northern Mozambique, which was included in previous surveys, was not included due to resource constraints.

As was done for prior surveys, we enrolled HIV-uninfected and HIV-infected children aged <5 years. HIV-uninfected children were enrolled in the community in Manhiça and were identified through age-stratified (1–11 months, 12–23 months, 24–59 months) random selection from the Manhiça Demographic Surveillance System (DSS) database [18]. Children were considered HIV-uninfected if HIV PCR or rapid test (for children >18 months of age) was negative or if they were born from a mother with a documented negative HIV test during pregnancy; HIV testing was offered for children who did not meet these criteria. If children and/or their guardians were not home when the field team arrived, visits were rescheduled via phone calls; a total of four visits were attempted before moving to the next child in the DSS list. HIV-infected children were enrolled as a convenience sample of patients presenting for routine care at outpatient HIV clinics Manhiça and Mavalane [17], stratified into 3 age groups: 1–11 months, 12–23 months and 24–59 months; enrollment for each age strata was stopped if the target sample size +20 % was reached in each age group. This approach was used starting with the pre-PCV survey in 2012–2013, when Mozambique had a high rate of mother-to-child transmission of HIV [19] and limited data were available on PCV impact among HIV-infected children. Over time the burden of pediatric HIV in Mozambique declined [20]; however, enrollment methods remained unchanged to ensure consistency with prior surveys.

For this survey, we expanded enrollment to include household members of all ages of the enrolled HIV-uninfected children with the aim of generating data that could serve as a baseline for assessing indirect effects of the changing PCV product (PCV10 to PCV13) and schedule (3 + 0 to 2 + 1). All household members of enrolled HIV-uninfected children present at the time of enrollment were also recruited, regardless of HIV status.

For all groups, only individuals without severe or acute respiratory illness at the time of enrollment were eligible for inclusion.

### 2.2 Sample size

Because the primary aim of this survey was to establish a new baseline for the subsequent evaluation of the PCV schedule and product changes in Mozambique, the sample size was based on feasibility; we planned to adjust the sample size for the post-policy-change surveys based on the prevalence of PCV10- and PCV13-unique type carriage in this survey. Considering the experience with enrollment with prior carriage surveys, declining rates of HIV infection in children, and plans to enroll HIV-infected children in only two sites (Maputo and Manhiça), the target enrollment numbers were 600 HIV-uninfected and 400 HIV-infected children aged <5 years. For the secondary objective of assessing whether PCV10-type carriage had further declined since the most recent survey (in 2015–2016), assuming α = 0.05 and power of 80 %, the target enrollment number would be sufficient to detect a reduction from 14.4 % to 10.5 % in HIV-uninfected and 22.6 % to 17 % in HIV-infected children. Sample size for household members was not calculated, since there was no planned comparison or required precision around estimates of carriage prevalence.

### 2.3 Data and sample collection

Study staff used a standardized questionnaire for each participant group to gather demographic data, household characteristics and other factors that may influence pneumococcal carriage. All clinical and demographic data were entered into an electronic device using an open-source, mobile data collection platform (ODK) and automatically uploaded to the study database built in Redcap Software for data cleaning. For individuals born after December 2012, HIV status and vaccination status were obtained from the child health card. Study staff also collected a single nasopharyngeal (NP) sample from each participant. NP samples were collected using sterile calcium alginate swabs (Puritan®-Calgiswab®, USA). After collection NP samples were immediately placed and stored in 1.0 mL of skim milk, tryptone-glucose-glycerol (STGG), prepared in-house. NP samples were sent to the laboratory within 4 h of collection at 4-8 °C and were vortexed for 10–20 s before storage at −70 °C [21,22].

### 2.4 Laboratory methods

For pneumococcus isolation, all NP samples underwent thawing, were vortexed for 10–20 s and were tested for culture bytransferring 200 μL of the specimen into 6.0 mL containing 0.5 % yeast extract (THY) combined with 1.0 mL of rabbit serum, both from Gibco™). After 6 h incubation at 37 °C+ 5 % of CO_2,_ 10 μL loop of broth growth were inoculated onto tryptone soy agar plates with 5 % sheep blood (BAP)). Alpha-hemolytic suspected pneumococcal colonies after overnight BAP incubation at 37 °C+ 5 % of CO_2_ were identified by optochin (BBL Taxo, Becton Dickinson) susceptibility and bile solubility (2 % sodium deoxycholate, Sigma-Aldrich, Steinhein, German) tests. When more than one potential pneumococcal colony morphology present per BAP, a representative of each colony type, were selected for further testing.

Pneumococcal isolates underwent serotyping by Quellung reaction. Quantitative polymerase chain reaction (qPCR) for pneumococcal *lyt*A gene detection was performed in all isolates that were non-typeable by Quellung reaction. All non-typeable *lyt*A positive isolates were submitted to serotyping by conventional multiplex polymerase chain reaction (cmPCR) according to the Centers for Disease Control and Prevention (CDC) protocol for multiplex PCR- *S. pneumoniae* serotyping [23,24].

Antimicrobial susceptibility testing was performed on pneumococcal isolates by broth microdilution (Sensititre Cation Adjusted Mueller-Hinton Broth with lysed horse blood, Thermo Scientific) to determine Minimal Inhibitory Concentration (MIC), using Thermo Scientific™ Sensititre™ Streptococcus STP6F antimicrobial susceptibility panel following manufacturer instructions and Sensititre auto-inoculator, under testing conditions recommended by Clinical and Laboratory Standards Institute (CLSI) guidelines. After incubation, MIC determination of each drug in the panel was performed visually using mirror magnification plate reader.

We assessed antimicrobial resistance patterns among the isolates for commonly used antibiotics. MIC results were categorized as susceptible, intermediate, or resistant according to the CLSI 2023 guidelines [25]. We examined both oral and parenteral non-meningitis breakpoints for penicillin.

### 2.5 Data analysis

We conducted descriptive analyses of characteristics of enrolled children disaggregated by HIV status. We also summarized characteristics of HIV-uninfected children's enrolled household members. We calculated overall and VT carriage prevalence, disaggregated by age group and HIV status. Based on preliminary comparisons of the randomly selected children aged <5 years and enrolled household members aged <5 years, there were no substantial differences in sex, age, PCV doses (Table S1) or distributions of serotypes by PCV formulation (Fig. S1); these two groups were therefore combined for all primary analyses.

We assessed changes in overall and VT prevalence (both PCV10 and PCV13-unique serotypes) among children aged <5 years between the third carriage study conducted in 2015–2016 (two-three years after PCV10 3 + 0 introduction) and the fourth conducted in 2018–2019 (five-six years after PCV10 3 + 0 introduction, mostly prior to the change to PCV13 and completely prior to the change to 3 + 0 schedule) for all children <5 and stratified by HIV status, using a chi-square test. We used log-binomial regression to calculate prevalence ratios and 95 % confidence intervals (overall and stratified by HIV status), adjusting for age category (<12 months, 12- < 24 months, 2- < 5 years), HIV status (overall only), and any (≥ 1) vs. none PCV10 vaccination (given the increased vaccination coverage between studies) to assess if PCV10 serotype carriage had significantly decreased. We also conducted a sensitivity analysis, restricting the 2018–2019 study to only HIV-positive children and the randomly selected HIV-negative children (i.e., excluding household members).

For the 2018–2019 carriage study, we described the distribution of individual serotypes and assessed distribution of serotypes by PCV formulation by age group and HIV status. We grouped serotypes into the following categories: PCV10 (1, 4, 5, 6B, 7F, 9 V, 14, 18C, 19F, 23F), PCV13 unique (3, 6 A, 19 A), PCV15 unique (22F, 33F), PCV20 unique (8, 10 A, 11 A, 12F, 15B), and non-vaccine type. Because of policy changes pertaining to both PCV10 and PCV13, individuals colonized with multiple serotypes were categorized as “PCV10” if any isolates were PCV10 and none were PCV13 unique, “PCV13 unique” if any isolates were PCV13 unique and none were PCV10, and “PCV10 and PCV13 unique” if an individual had both a PCV10 and a PCV13 unique isolate. For higher valent PCV serotypes, we used a hierarchical approach; for individuals without any PCV10 or PCV13 unique isolates, we categorized them as “PCV15 unique” if they had a PCV15 unique isolate but no PCV20 unique serotype and as “PCV20 unique” if they had at least one PCV20 unique isolate. Colonized individuals without any vaccine serotype, including those with a non-typable isolate, were classified as “non-vaccine type”. Nineteen samples without any pneumococcal growth detected were considered negative. We also analyzed the distribution of individual serotypes among all isolates from the study.

We analyzed the serotypes among colonized adult household members of colonized children under five, assessing if they were the same or different as the serotypes among the children under five in the household to identify potential transmission. We conducted a risk factor analysis for overall colonization in adult (aged 18 years and older) household members, using generalized estimating equation modeling to account for household clustering. We used a stepwise model selection process based on Akaike information criterion to determine which demographic and household variables to include in the model.

### 2.6 Ethical considerations

Informed written consent was obtained from the parent or guardian for all children aged <12 years. For participants aged 12–17 years, study staff obtained informed written assent from the participants in addition to consent from a parent or guardian. For participants aged ≥18 years, study staff obtained informed consent from the participants. The study was reviewed and approved by the CISM scientific committee, National Committee for Bioethics and Health in Mozambique, and the Centers for Disease Controls and Prevention's Institutional Review Board.^3^

## 3 Results

### 3.1 Study population characteristics

**Fig. 1:**
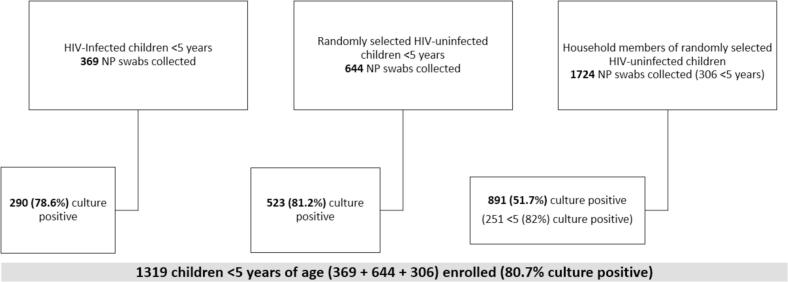
Study population October 2018 – June 2019, Mozambique.

We enrolled 369 HIV-infected children from clinics in Mavalane and Manhiça, 644 randomly selected HIV-uninfected children from the community in Manhiça, and 1724 of their household members (including 306 aged <5 years) (Fig. 1). Enrolled HIV-infected and HIV-uninfected children were similar with regard to age distribution, nutritional status (based on weight-for-length/height z-score), household size, and presence of smokers in the household (Table 1). HIV-infected children (recruited in both rural and urban sites) more frequently reported school or childcare attendance, antibiotic use, and electricity in the household than HIV-uninfected children (enrolled only in rural site). A median of 50 % of household members of randomly selected children in Manhiça were enrolled; 9 % of household members who were approached refused to enroll in the study, and the remaining members were not home at time of enrollment. One-third (32.3 %) of enrolled household members were male, and the median age was 15 years (Table 2).

**Table 1:** Individual and household characteristics of all children under five by HIV status – Mozambique, 2018–2019.

	HIV-infected (*n* = 373)+ n (%)	HIV-uninfected (*n* = 910)^++^ n (%)	HIV status unknown (*n* = 36)^+++^ n (%)
Individual characteristics			
Male	191 (51.2 %)	464 (51 %)	18 (50 %)
Median age, years (IQR)	2 (1,3)	2 (1, 3)	3 (2, 4)
< 12 months	72 (19.3 %)	167 (18.4 %)	1 (2.8 %)
12–23 months	88 (23.6 %)	247 (27.1 %)	3 (8.3 %)
≥24 months	213 (57.1 %)	496 (54.5 %)	32 (88.9 %)
Location			
Maputo HIV clinic	205 (55 %)	NA	NA
Manhiça HIV clinic	164 (44.4 %)	NA	NA
Manhiça community	4 (1.1 %)	910 (100 %)	36 (100 %)
Median weight-for-length/height z-score (IQR)	0.40 (−0.72, 1.41)	0.35 (−0.68, 1.25)	NA
PCV doses⁎			
0	19 (6.5 %)	5 (0.6 %)	0
1	12 (4.1 %)	12 (1.4 %)	0
2	11 (3.8 %)	22 (2.6 %)	1 (6.3 %)
3	250 (85.6 %)	797 (95.3 %)	15 (93.8 %)
Child in school or childcare center	28 (7.5 %)	31 (3.4 %)	NA
Self-reported anti-microbial use within 2 weeks prior to swab collection⁎⁎			
Yes	163 (44.2 %)	41 (6.5 %)	0
No	206 (55.8 %)	603 (93.6 %)	0
HIV exposure (among HIV-uninfected)	NA	153 (23.8 %)±	NA
On antiretroviral therapy (among HIV-infected)	355 (95.2 %)	NA	NA
Household characteristics⁎⁎⁎			
Median number of household members participating in carriage study (IQR)	NA	2 (1, 4)	
Median number of household members including reference child (IQR)	5 (4, 7)	5 (4, 7)	
Median number of rooms in house	2 (1, 3)	2 (1,2)	
Anyone in family smokes	47 (12.7 %)	70 (10.9 %)	
Household has electricity	284 (77.0 %)	290 (45.0 %)	

HIV = human immunodeficiency virus; IQR = Interquartile range.

+ 4 from household members; ^++^ 266 from household members, ^+++^ all from 36 household members.

⁎ 81, 74, and 20 HIV-infected, HIV-uninfected, unknown HIV status children, respectively, missing PCV dose information.

⁎⁎ 4, 266, and 36 HIV-infected, HIV-uninfected, unknown HIV status children, respectively, missing antibiotic information.

⁎⁎⁎ HIV-infected children come from both urban and rural settings, while HIV-uninfected come from a rural setting only.

± data available only for randomly selected HIV-uninfected only.

**Table 2:** Individual characteristics of household members of children randomly selected for enrollment from the community in Manhiça.

	N (%) (*N* = 1724)
Male	556 (32.3 %)
Median age (IQR)	15 (6, 28)
<5 years	306 (17.8 %)
5- < 18 years	614 (35.6 %)
18+ years	804 (46.6 %)
3 doses of PCV10	
Children <5, *N* = 306	235 (76.8 %)
Children 5- < 18 years, *N* = 614⁎	19 (3.1 %)
HIV status	
Positive	177 (10.3 %)
Negative	1104 (64.0 %)
Unknown	443 (25.7 %)
Median % household members enrolled (IQR)	50 % (33 %, 60 %)

IQR = interquartile range.

⁎ Vaccination status unknown for 88.1 % of children 5- < 18 years.

### 3.2 Overall and vaccine-type carriage in 2018–2019 carriage study

**Fig. 2:**
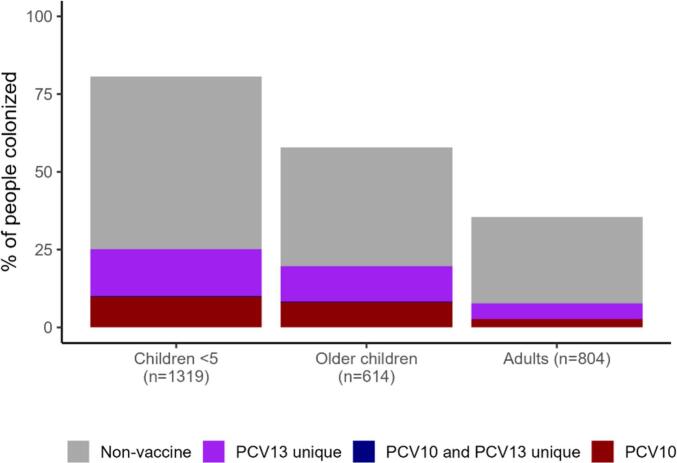
Distribution of serotypes by PCV formulation and age group among children <5 years old with pneumococcal carriage, 2018–2019.

**Fig. 3:**
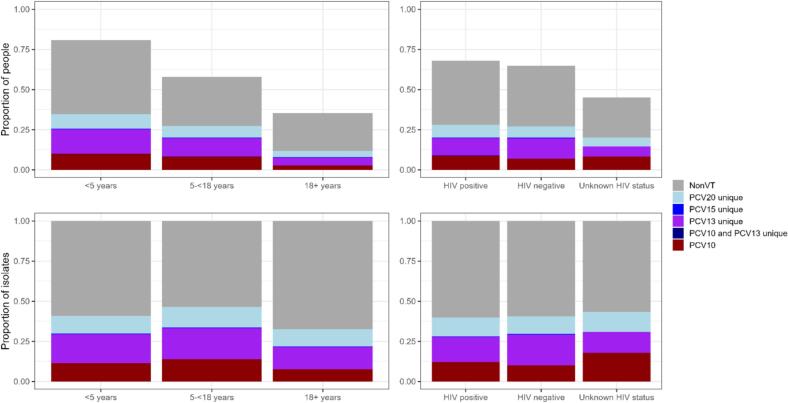
Distribution of serotypes by PCV formulation by age group and HIV status among all individuals enrolled in 2018–2019 carriage study (top row) and among all colonized individuals (bottom row).

Among all 1319 children aged under five years enrolled in 2018–2019 (369 HIV-infected children, 644 randomly selected HIV-uninfected children, 306 household members of the randomly selected children), 1064 (80.7 %) were colonized with pneumococcus (Fig. 2), including 87 with 2 distinct (i.e., different serotypes) isolates, and 3 with 3 distinct isolates (total of 1157 isolates). Among 614 children aged 5- < 18 years, 355 (57.8 %) were colonized (Fig. 2), including 16 with 2 distinct isolates (total of 371 isolates). Among 804 adults (aged ≥18 years), 285 (35.4 %) were colonized, including 4 with 2 distinct isolates (total of 289 isolates). Non-vaccine and PCV13-unique serotypes were more common than PCV10 serotypes among all age groups. The distribution of serotypes by PCV formulation among colonized individuals was similar across age groups (first panel of Fig. 3 and Fig. S2) and HIV status (second panel of Fig. 3).

### 3.3 Serotype distribution

**Fig. 4:**
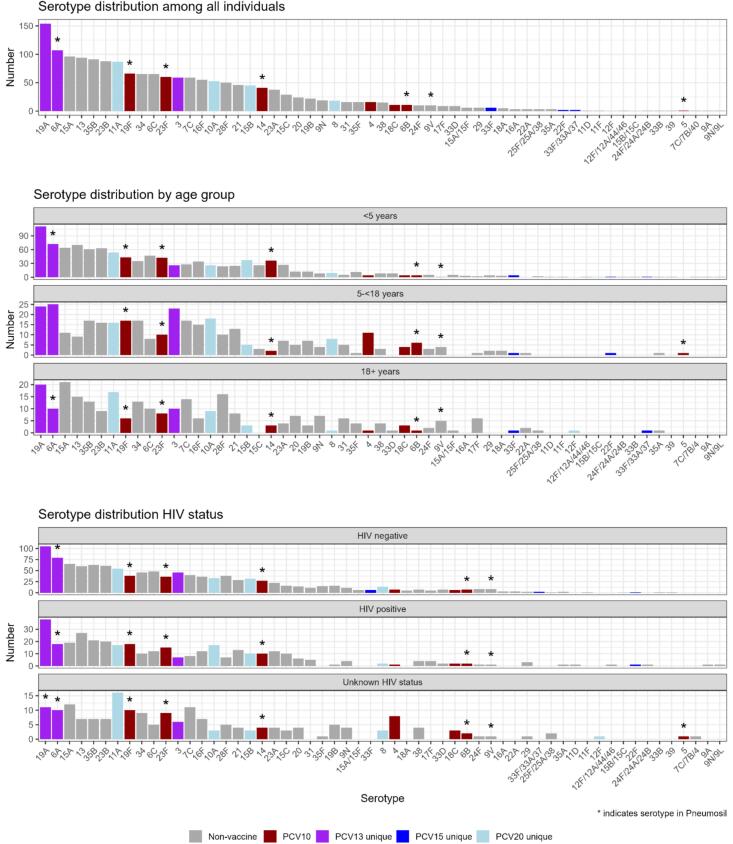
Individual serotype distribution* for all colonized individuals enrolled in 2018–2019 carriage study.

Among 1817 total isolates collected in 2018–2019, the most frequently observed serotypes were 19 A (*n* = 154, 8.5 % of isolates) and 6 A (*n* = 107, 5.9 %), which are both PCV13-unique serotypes and covered by PCV10-SII (Fig. 4). PCV10 serotypes 19F (*n* = 66, 3.6 %) and 23F (*n* = 60, 3.3 %) were among the most common serotypes. PCV20 unique serotypes (*n* = 204, 11.2 %) were more common than PCV15 unique serotypes (*n* = 8, 4.4 %). We observed similar patterns across age groups, although serotypes 3 (PCV13 unique), 4 (PCV10), and 6B (PCV10) were more common among 5- < 18 year olds than other age groups (Fig. S3).

### 3.4 Risk factors for adult colonization

Among 285 colonized adults, 257 (90.2 %) lived in a household with at least one colonized child aged <5 years. Among the 250 typeable isolates from these colonized adults residing in a household with a colonized child, 127 (50.1 %) were a serotype that was also carried by a child in their household. Among adults, significant risk factors for colonization included the number of colonized children under five in the household (1.62 times the odds of an adult being colonized for each additional colonized child under five) and female sex (1.53 time the odds compared to male sex) (Table 3).

**Table 3:** Risk factors for pneumococcal colonization in adult (aged 18 and older) household members (*n* = 804) for 2018–2019 carriage study.

Variable	Colonized *n* = 285 n (%) or median (IQR)	Not colonized *n* = 519 n (%) or median (IQR)	Crude odds ratio (95 % bounds)	Adjusted odds ratio (95 % bounds)
Age in years	28 (21, 35)	30 (23, 39)	0.99 (0.98–1.00)	0.99 (0.98–1.00)
Female	245 (86.0)	412 (79.4 %)	1.59 (1.07–2.35)⁎	1.53 (1.02–2.30)⁎
Number of household members	6 (4, 7)	6 (4, 8)	0.99 (0.94–1.04)	0.94 (0.89–1.00)
Number of colonized children aged under 5 years in household	1 (1, 2)	1 (1, 1.5)	1.53 (1.24–1.89)⁎	1.62 (1.30–2.02)⁎

IQR = interquartile range.

⁎ significant (*p* value <0.05).

### 3.5 Antimicrobial resistance

**Fig. 5:**
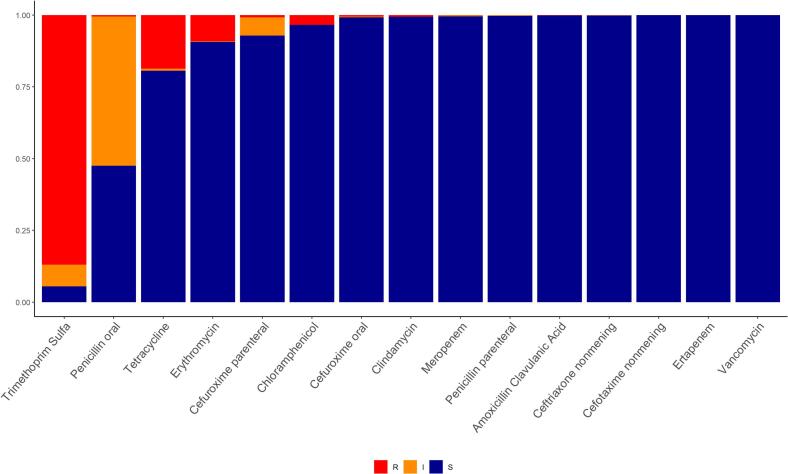
Pneumococcal AMR patterns among isolates* from the 2018–2019 carriage study, grouped into susceptible (S), intermediate (I), resistant (R). * *n* = 1802 isolates for all except for Clindamycin (*n* = 1798).

Among pneumococcal isolates, non-susceptibility (including resistant and intermediate) was most frequently observed for trimethoprim-sulfa and oral penicillin (Fig. 5). Similar trends across age groups (Fig. S4) and HIV status (Fig. S5) were observed, with slightly lower susceptibility among HIV-infected children (Fig. S5).

### 3.6 Overall and vaccine-type carriage in children aged under five years over time

**Fig. 6:**
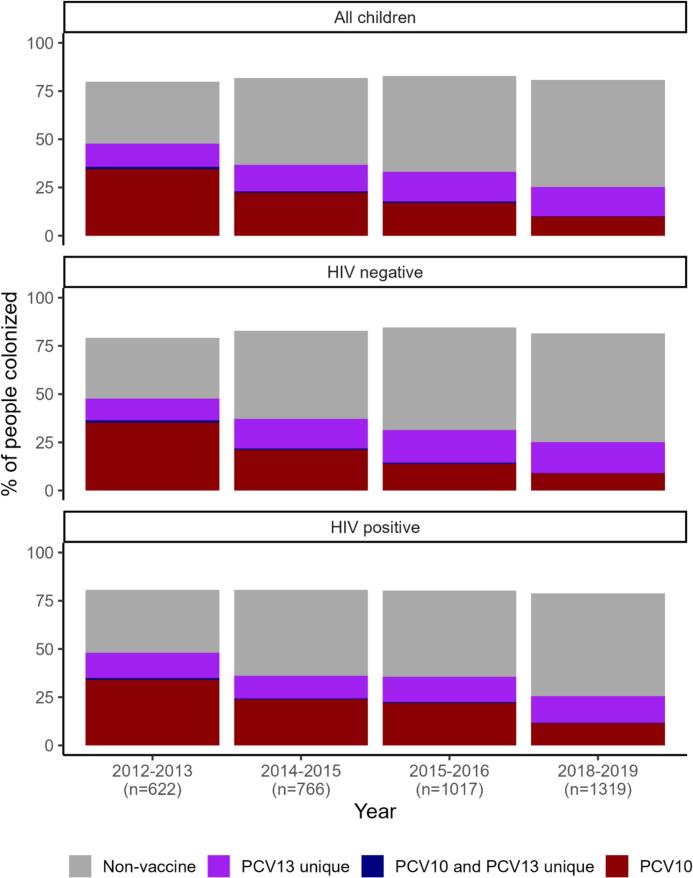
Carriage over time by distribution of serotypes by PCV formulation among children aged <5 years and by HIV status.

Overall carriage prevalence remained stable at approximately 80 % across the four carriage studies conducted between 2012 and 2019 (Fig. 6), with similar patterns when disaggregated by finer age groups (Fig. S6). Between 2012 and 2013 (prior to PCV10 3 + 0 introduction) and 2015–2016 (two-three years after PCV10 3 + 0 introduction), the prevalence of PCV10 type carriage declined from 35.7 % to 17.7 %. Between 2015 and 2016 and 2018–2019 (five-six years after PCV10 3 + 0 introduction, mostly prior to the change to PCV13 and completely prior to the change to 3 + 0 schedule), the prevalence further declined from 17.7 % to 10.1 % among all children <5 years, reflecting a significant reduction (chi-square test *p*-value <0.001), with an adjusted prevalence ratio of 0.72 (95 % confidence interval 0.41–1.27) (Table S2). Among HIV-positive children <5 years, the prevalence declined from 22.6 % to 11.8 % (*p* < 0.001), with an adjusted prevalence ratio of 0.58 (95 % confidence interval 0.32–1.04) and among HIV-negative children <5 years, the prevalence declined from 14.3 % to 9.1 % (*p* = 0.002), with an adjusted prevalence ratio of 0.82 (95 % confidence interval 0.67–1.0) (Table S2). Our sensitivity analysis excluding household members found nearly identical prevalence ratios (Table S2). The prevalence of carriage of non-PCV10 vaccine serotypes (i.e., non-vaccine and PCV-13 unique serotypes) increased over the four carriage studies from 44.2 % to 70.6 % (p < 0.001) among all children <5 years, from 45.7 % to 67.0 % (p < 0.001) among HIV-infected children <5 years, and from 42.7 % to 72.3 % (p < 0.001) among HIV-uninfected children <5 years. The prevalence of carriage of PCV13-unique serotypes increased over the four carriage studies from 12.1 % to 15.1 % (*p* = 0.09) among all children, from 13.0 % to 13.7 % (*p* = 0.89), among HIV-infected children, and from 11.1 % to 16.4 % (*p* = 0.04) among HIV-uninfected children (Fig. 6).

## 4 Discussion

In southern Mozambique, where pneumococcal vaccine-type carriage among children aged <5 years had declined by 50 % within three years after PCV10 introduction, we found further reduction between years three to five post-introduction, suggesting that the benefits of PCV10 introduction were still accumulating. However, even after five years of PCV10 use in a high coverage (>90 % in study population) setting, approximately one in ten children aged under five years, and one in twenty persons aged five years or older, were colonized with a PCV10 serotype, reflecting continued circulation of vaccine serotypes. Carriage of PCV13-unique serotypes increased during this time, and serotypes 19 A and 6 A were the most commonly detected. These findings underscore the potential impact of the recent change from PCV10 to PCV13 formulation and the schedule change.

The persistence of vaccine-type carriage even in the setting of mature PCV programs has been reported in other contexts in sub-Saharan Africa and Asia, including PCV10 [16,[26], [27], [28]] and PCV13 [[29], [30], [31], [32]] using countries. Several other sub-Saharan African countries experienced similar initial declines in vaccine-type carriage in the first few years after vaccine introduction, followed by a levelling off [29,33,34]. In Mozambique, we observed some additional decreases in PCV10 serotype carriage between years three to five years post-PCV introduction among both HIV-uninfected and –infected children; however, the adjusted prevalence ratios were not statistically significant.

One strategy proposed for more optimal control of vaccine-type circulation in high-burden settings is to change to a booster dose containing schedule [13], and Mozambique switched the PCV schedule from 3 + 0 to 2 + 1 in 2019, soon after this study was conducted. The rationale behind the schedule change is that higher levels of immunity may be sustained for longer when a booster dose is given, which could lead to stronger direct and indirect effects in the community. Evidence to support this comes from South Africa, which introduced PCV with a 2 + 1 schedule in 2009 and has seen more robust direct and indirect effects, including against serotype 1, which causes substantial morbidity and mortality, compared to many countries that implemented a 3 + 0 schedule [35,36]. The data from this study, which examines carriage among older children and adults for the first time in this context, will serve as a baseline for assessing the impact of the schedule change on PCV10 carriage among children and adults.

These data will also serve as a baseline for analyzing the potential impact of different PCV formulations, including PCV13, which was introduced near the end of this study in southern Mozambique, PCV10-SII, and extended-valency vaccines now available in high income countries (i.e., PCV15 and PCV20). Serotypes 19 A and 6 A, which are included in PCV13 and PCV10-SII but not in PCV10, were the two most frequently detected serotypes across all age groups in this study. Serotype 3, the other PCV13-unique serotype, was also relatively common. PCV13 introduction has led to reductions in PCV13-type carriage in several sub-Saharan African countries, including the Gambia [37], Burkina Faso [38], Cameroon [39], South Africa [40], and Zimbabwe [41]. Recent studies suggest PCV13 has provided both direct and indirect protection against serotype 3 [42]. As we found and has been found elsewhere [27,43], when vaccine serotypes decline, they are replaced by serotypes not in the vaccine, keeping overall colonization rates stable. For example, the PCV20 serotype 10 A was among those contributing to the increase in non-vaccine serotypes in this study, which is a trend that has been observed elsewhere, both in carriage and invasive pneumococcal disease serotype distribution [44,45]. This finding has potential implications for future vaccine policy. However, 15- and 20- valent PCVs are not yet available in low-income countries such as Mozambique.

Higher valent vaccines include serotypes that tend to be highly resistant to commonly used antimicrobials, including serotype 19 A. The switch from PCV10 to PCV13 therefore has the potential to reduce carriage of resistance. Compared with the data from most recent carriage study carried out in Mozambique (in 2015–2016) [12], antimicrobial susceptibility patterns in carriage have remained generally stable. Our findings underscore the high prevalence of pneumococcal isolates resistance to trimethoprim sulfamethoxazole and penicillin; this result aligns with previous carriage studies carried out in our study area, where similar resistance patterns were observed [12]. Penicillin-resistant *S. pneumoniae* is one of the World Health Organization's top pathogens for concern with regard to antimicrobial resistance, so continued monitoring of these trends through studies like ours will be critical for informing global efforts [46]. Resistance to trimethoprim sulfamethoxazole may be driven by its wide use as a prophylaxis for HIV-infected individuals; prophylaxis may lead to selection and amplification of a trimethoprim sulfamethoxazole resistance specific gene, which contributes to the increase and spread of resistant strains among pneumococcal carriage groups [47,48].

Relatively limited data are available on risk factors for pneumococcal carriage among adults in high-burden settings [[49], [50], [51]]. As expected, a high proportion of serotypes among adult household members was the same as the serotype of the household's colonized child under five, indicating potential transmission. The number of colonized children under five in a household was a significant risk factor for colonization among adults. In addition, being female was a risk factor, which aligns with other studies that have found the primary caretakers of children, who are often female, to be at higher risk [52,53].

This study is subject to several limitations. Household members were a random sample from households with HIV uninfected children under five, as opposed to a pure random sample of adults; this means the results of this study are generalizable to households with an HIV-uninfected child under five as opposed to all households. In addition, HIV-uninfected children were randomly sampled from a list of children, meaning houses with more children under five were more likely to be included. As discussed above, we analyzed the randomly selected children under five together with the household members under five because we did not observe any meaningful differences in these groups and because all HIV-negative household members under five were eligible to be included as reference children. However, it is possible these groups could differ in ways we did not observe. Because the HIV-infected children were recruited from routine care clinics, they likely do not represent all HIV-infected children. Furthermore, some of the older children (up to 10 or 11 years old) were fully vaccinated with PCV10, so the household members' data reflected both a combination of direct and indirect effects. Finally, we did not have data on whether any of the children had received PCV13; however, given the timeline of the formulation change in the study area (starting with new birth cohorts in May 2019) relative to the carriage study (October 2018 to July 2019), no children in the study would have received the full series with PCV13.

Despite these limitations, the data from this study have the potential to serve as an important baseline for assessing the impact of recent and future policy changes. In addition to providing evidence of continued declines, yet persistent carriage of, PCV10 serotypes in children under five, this study provides the first insights into carriage among older children and adult household members of these children. These data will be critical for assessing the indirect effects of the PCV13 formulation and schedule changes. The findings from future studies, which will use this study as a baseline, will have important implications for global pneumococcal vaccination policy.

## Disclaimer

The findings and conclusions in this report are those of the authors and do not necessarily represent the official position of the U.S. Centers for Disease Control and Prevention.

## Funding

This work was supported by the 10.13039/100000865Bill & Melinda Gates Foundation (INV-006502).

*112 nontypeable pneumococcal isolates excluded.

## Credit authorship contribution statement

**Rebecca Kahn:** Writing – original draft, Visualization, Software, Methodology, Formal analysis. **Benild Moiane:** Writing – original draft, Formal analysis, Data curation. **Fernanda C. Lessa:** Writing – review & editing, Supervision, Data curation, Conceptualization. **Sergio Massora:** Writing – review & editing, Supervision, Data curation, Conceptualization. **Viviana Mabombo:** Writing – review & editing, Methodology, Data curation. **Alberto Chauque:** Writing – review & editing, Formal analysis, Data curation. **Nelson Tembe:** Writing – review & editing, Methodology, Data curation, Conceptualization. **Helio Mucavele:** Writing – review & editing, Methodology, Data curation, Conceptualization. **Cynthia G. Whitney:** Writing – review & editing, Methodology, Conceptualization. **Charfudin Sacoor:** Writing – review & editing, Methodology, Data curation, Conceptualization. **Graca Matsinhe:** Writing – review & editing, Methodology, Data curation, Conceptualization. **Fabiana C. Pimenta:** Writing – review & editing, Methodology, Formal analysis, Data curation, Conceptualization. **Maria da Gloria Carvalho:** Writing – review & editing, Supervision, Resources, Methodology, Formal analysis, Data curation, Conceptualization. **Betuel Sigauque:** Writing – review & editing, Supervision, Methodology, Data curation, Conceptualization. **Jennifer Verani:** Writing – review & editing, Supervision, Project administration, Methodology, Conceptualization.

## Declaration of competing interest

The authors declare the following financial interests/personal relationships which may be considered as potential competing interests:

Support for project reports financial support was provided by the 10.13039/100000865Bill & Melinda Gates Foundation (INV-006502). If there are other authors, they declare that they have no known competing financial interests or personal relationships that could have appeared to influence the work reported in this paper.

## Data Availability

The data that has been used is confidential.

## Appendix A Supplementary data

Supplementary material 1 Image 1

Supplementary material 2 Image 2

## Footnotes

3 ^§^ See 45C.F.R. part 46; 21C.F.R. part 56
