# Corrigendum to “Nasopharyngeal carriage of *Streptococcus pneumoniae* among children and their household members in southern Mozambique five years after PCV10 introduction” Vaccine, Volume 47, 15 February 2025, 126,691

**DOI:** 10.1016/j.vaccine.2025.127124

**Published:** 2025-05-22

**Authors:** Rebecca Kahn, Benild Moiane, Fernanda C. Lessa, Sergio Massora, Viviana Mabombo, Alberto Chauque, Nelson Tembe, Helio Mucavele, Cynthia G. Whitney, Charfudin Sacoor, Graca Matsinhe, Fabiana C. Pimenta, Maria da Gloria Carvalho, Betuel Sigauque, Jennifer Verani

**Affiliations:** a Respiratory Diseases Branch, Division of Bacterial Diseases, Centers for Disease Control and Prevention, Atlanta, United States; b Centro de Investigação em Saúde da Manhiça (CISM), Maputo, Mozambique; c International Infection Control Branch, Division of Healthcare Quality Promotion, Centers for Disease Control and Prevention, Atlanta, United States; d Instituto Nacional de Saúde, Ministério de Saúde, Maputo, Mozambique; e Emory University, Atlanta, United States; f John Snow Inc. (JSI) on the MOMENTUM Routine Immunization Transformation and Equity (M-RITE), Maputo, Mozambique

The authors regret that the fig. 2 title was incorrect. It should read “Fig. 2. Distribution of serotypes by PCV formulation and age group, 2018–2019”.

The authors would like to apologise for any inconvenience caused.

