# Supplementary material for: Nasopharyngeal carriage of Streptococcus pneumoniae among children and their household members in southern Mozambique five years after PCV10 introduction
Source: Vaccine. 2025 Feb 15;47:None. doi: 10.1016/j.vaccine.2024.126691 (PMC11797556; doi:10.1016/j.vaccine.2024.126691)

Supplement for Nasopharyngeal carriage of *Streptococcus pneumoniae* among children and their household members in Southern Mozambique 5 years after PCV-10 introduction

Table S1: Comparison of HIV-uninfected children aged <5 years randomly selected from Manhiça DSS database and their household members aged <5 years

	**Children aged <5 years randomly selected (n=644) n (%)**	**Household members aged <5 years (n=306) n (%)**
Male sex	342 (53.1%)	142 (46.4%)
Median age (IQR)	2 (1, 3)	2 (1, 4)
< 12 months	127 (19.7%)	41 (13.4%)
12 – 23 months	181 (28.1%)	69 (22.5%)
≥24 months	336 (52.2%)	196 (64.1%)
PCV doses*		
0	2 (0.3%)	3 (1.2%)
1	8 (1.3%)	4 (1.6%)
2	18 (3.0%)	5 (2.0%)
3	581 (95.4%)	223 (95.1%)

IQR = interquartile range

*missing for 35 children randomly selected and 61 household members

DSS= Demographic Surveillance System

Table S2: Prevalence ratios for PCV10 carriage from 2018-2019 to 2015-2016 studies

	All children <5	HIV-negative children <5	HIV-positive children <5	All children <5 excluding household members who were not randomly selected
PCV10 prevalence ratio comparing 2018-2019 to 2015-2016 (unadjusted)	0.57 (0.46-0.70)	0.63 (0.48-0.84)	0.52 (0.38-0.73)	0.58 (0.46 – 0.73)
PCV10 prevalence ratio comparing 2018-2019 to 2015-2016 (adjusted)	0.72 (0.41-1.27)*	0.82 (0.67-1.0)**	0.58 (0.32-1.04)**	0.71 (0.40-1.24)*

*Adjusted for HIV status, age category, PCV10 (any vs. no doses)

** Adjusted for age category and PCV10 (any vs. no doses)

Figure S1: Carriage by distribution of serotypes by PCV formulation comparing the randomly selected HIV-uninfected children under fiveto their household members under five

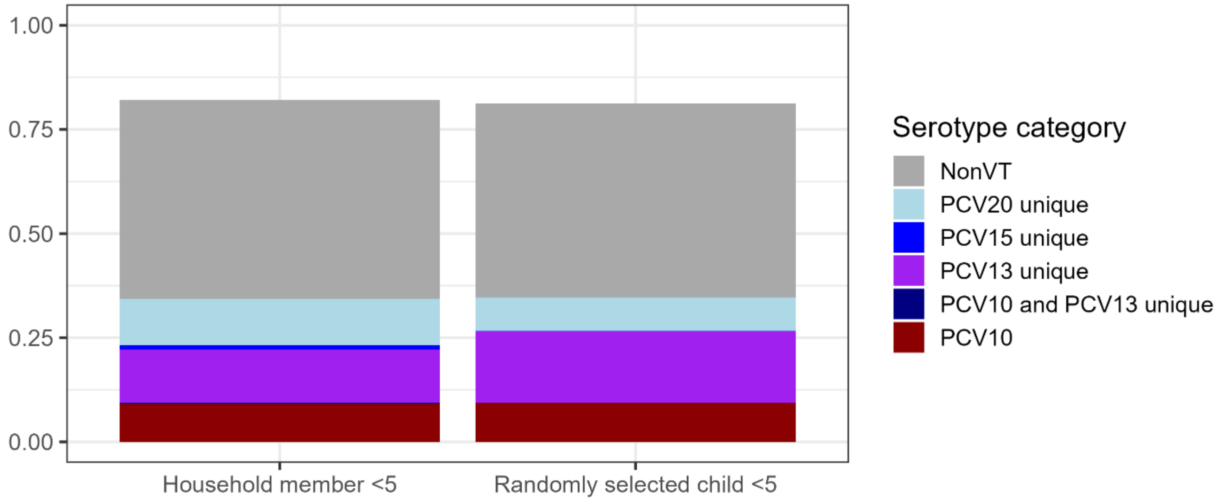

Note: 36 household members with unknown HIV status from the 2018-2019 study excluded

Figure S2: Serotype category distribution by finer age groups

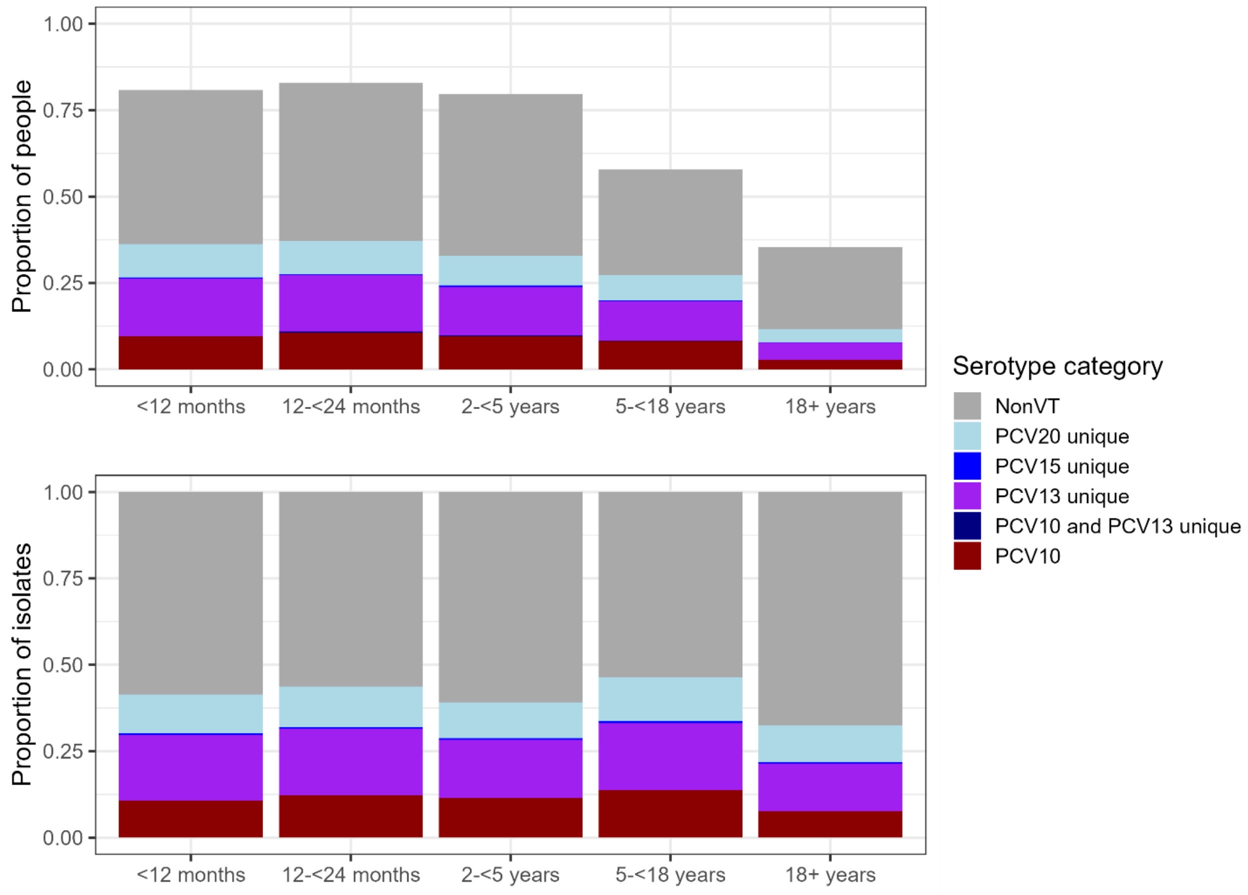

Figure S3: Serotype distribution by age

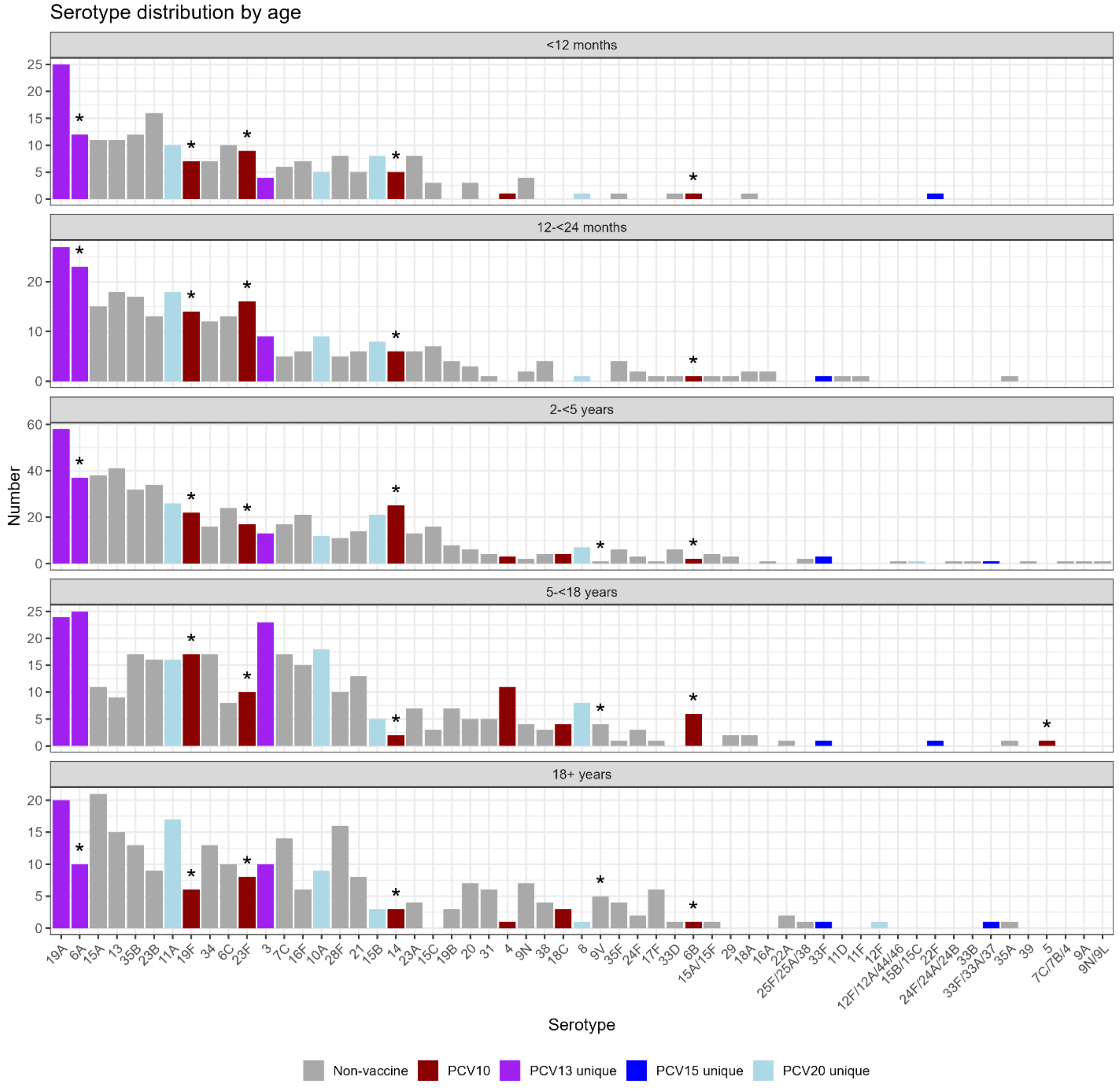

* indicates serotype in Pneumosil

Figure S4: Resistance patterns by age

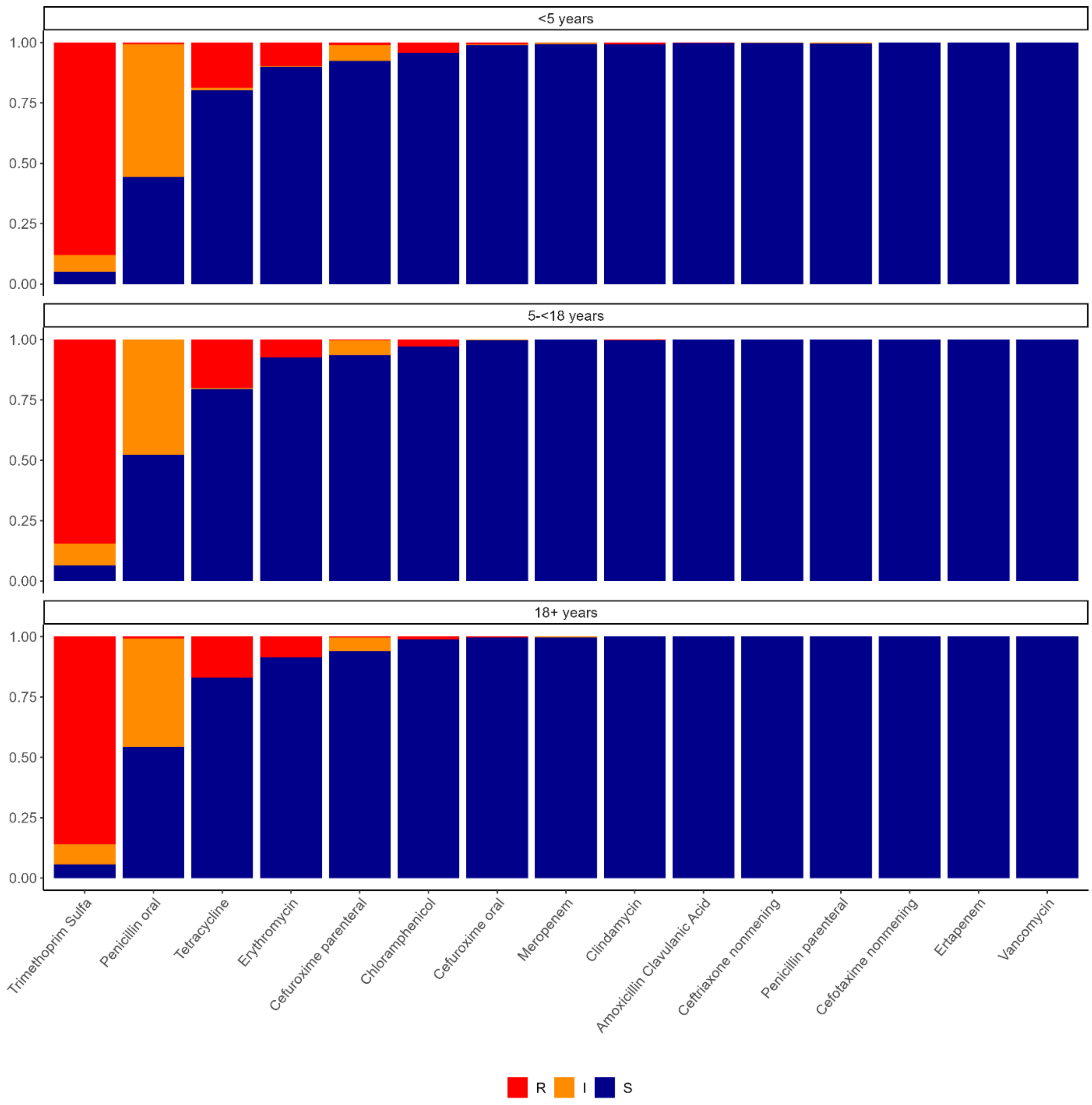

Figure S5: Resistance patterns by HIV status

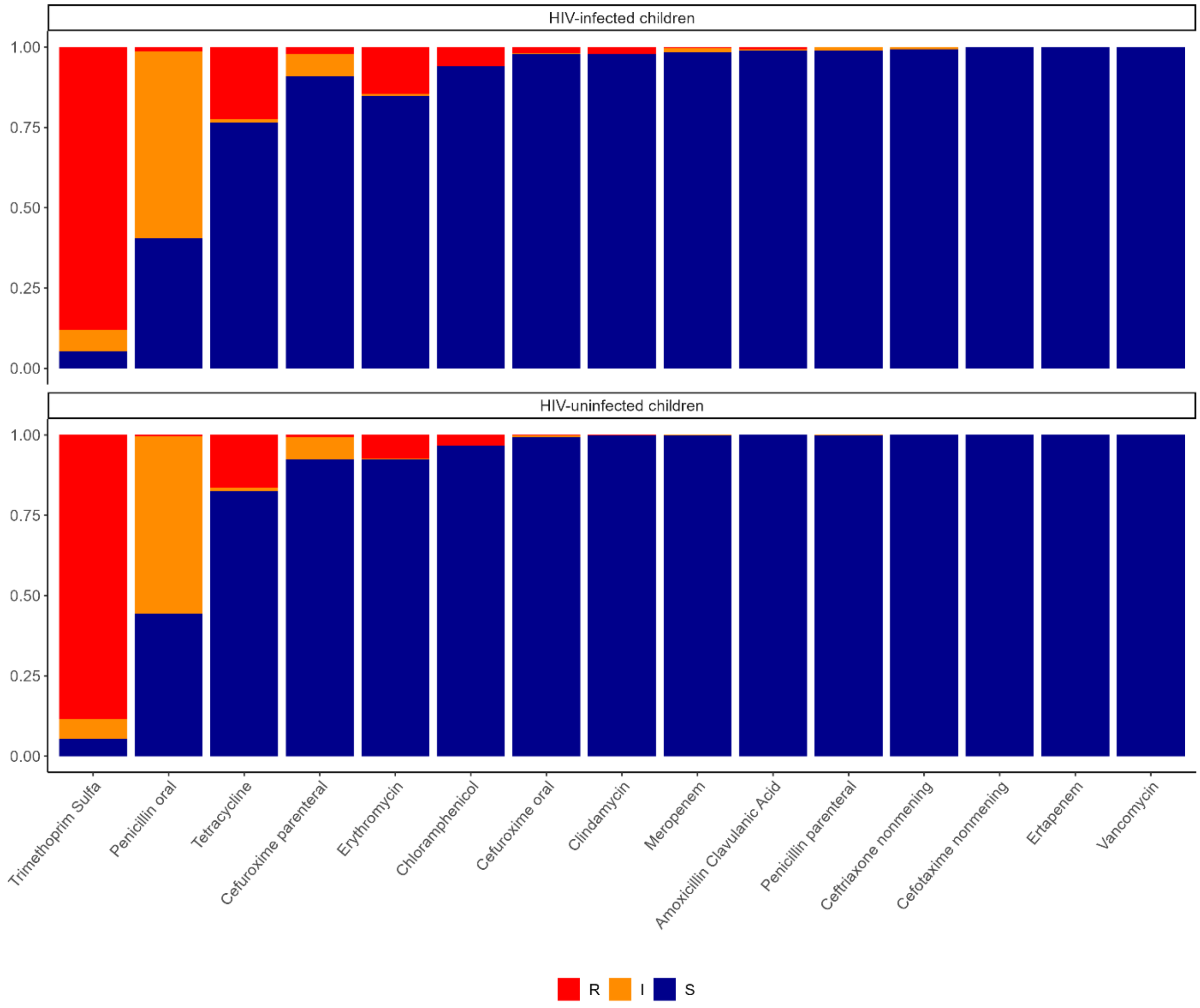

Figure S6: Carriage over time among children under five, disaggregated by age

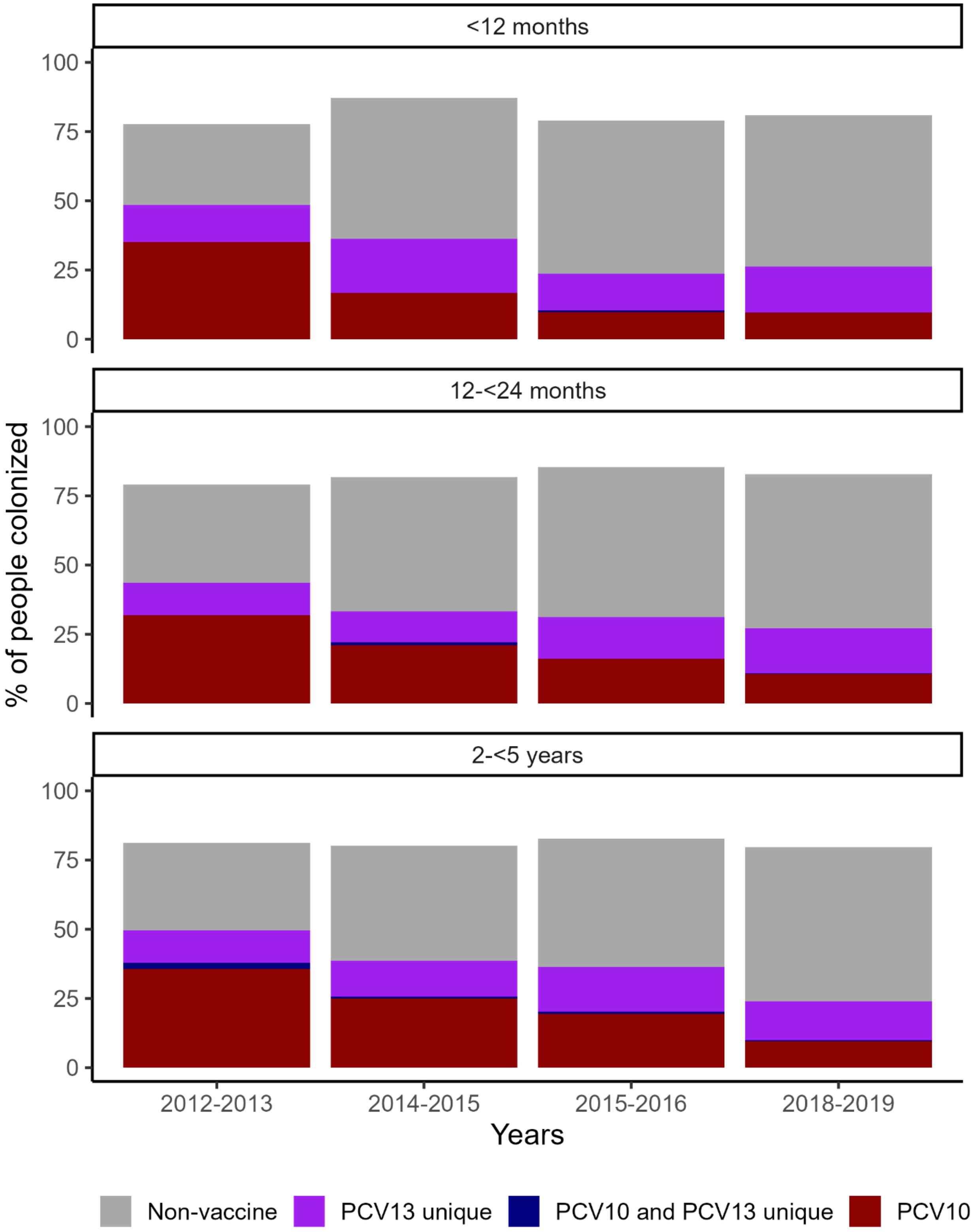

Supplement: Supplementary file 2 — Supplementary material 2 [file mmc2.pdf]
